# Production of enriched in B vitamins biomass of Yarrowia lipolytica grown in biofuel waste

**DOI:** 10.1016/j.sjbs.2021.02.027

**Published:** 2021-02-19

**Authors:** Monika Elżbieta Jach, Ewa Sajnaga, Monika Janeczko, Marek Juda, Elżbieta Kochanowicz, Tomasz Baj, Anna Malm

**Affiliations:** a Department of Molecular Biology, The John Paul II Catholic University of Lublin, 1I Konstantynów Street, 20-708 Lublin, Poland; b Laboratory of Biocontrol, Application and Production of EPN, Centre for Interdisciplinary Research, The John Paul II Catholic University of Lublin, 1J Konstantynów Street, 20-708 Lublin, Poland; c Department of Pharmaceutical Microbiology, Medical University of Lublin, 1 Chodzki Street, 20-093 Lublin, Poland; d Chair and Department of Pharmacognosy, Medical University of Lublin, 1 Chodzki Street, 20-093 Lublin, Poland

**Keywords:** B vitamins, Biotin, Biofuel waste, Folic acid, Oleaginous yeast, Pyridoxine, Thiamine, Riboflavin, Yarrowia lipolytica

## Abstract

*Yarrowia lipolytica* as an oleaginous yeast is capable of growing in various non-conventional hydrophobic substrate types, especially industrial wastes. In this study, the content of thiamine (vitamin B1), riboflavin (vitamin B2), pyridoxine (vitamin B6), biotin (vitamin B7) and folic acid (vitamin B9) in the wet biomass of *Y. lipolytica* strains cultivated in biofuel waste (SK medium), compared to the standard laboratory YPD medium, was assessed. Additionally, the biomass of *Y. lipolytica* A-101 grown in biofuel waste (SK medium) was dried and examined for B vitamins concentration according to the recommended microbial methods by AOAC Official Methods. The mean values of these vitamins per 100 g of dry weight of *Y. lipolytica* grown in biofuel waste (SK medium) were as follows: thiamine 1.3 mg/100 g, riboflavin 5.3 mg/100 g, pyridoxine 4.9 mg/100 g, biotin 20.0 µg/100 g, and folic acid 249 µg/100 g. We have demonstrated that the dried biomass is a good source of B vitamins which can be used as nutraceuticals to supplement human diet, especially for people at risk of B vitamin deficiencies in developed countries. Moreover, the biodegradation of biofuel waste by *Y. lipolytica* is desired for environmental protection.

## 1 Introduction

Oleaginous yeast *Yarrowia lipolytica* is well-known for its ability to grow in a wide range of substrates, especially non-conventional hydrophobic ones, such as vegetable or animal-waste fats, different fractions of petroleum, or waste streams from various industries (Dourou et al., 2018, Groenewald et al., 2013, Jach et al., 2017, Jach and Serefko, 2018, Katre et al., 2012, Lopes et al., 2018, Lopes et al., 2019, Papanikolaou et al., 2001, Papanikolaou et al., 2003, Rywińska et al., 2013). The fatty waste biodegradation by this yeast is very important for environmental protection in line with the take-make-dispose concept (Katre et al., 2012, Lopes et al., 2019, Saygün et al., 2014, Thevenieau et al., 2010, Vasiliadou et al., 2018, Tzirita et al., 2018). Huge amounts of the biodiesel production generate large quantities of residues or by-products which cannot be utilized in biodiesel production process (Eliche-Quesada et al., 2012). Due to the environmental problems associated with this waste, the recycling and valorization of these pollutants residues becomes urgent. One way to recycle fatty waste is its utilization as fermentation medium component for the production of added value compounds by microorganisms like yeast (Lopes et al., 2019). Thus, the ability of *Y. lipolytica* to produce biomass rich in various nutritional components in available inexpensive oily wastes, as carbon sources, is highly beneficial for protecting the environment (Dobrowolski et al., 2016, Groenewald et al., 2013, Jach et al., 2017, Jach and Serefko, 2018, Katre et al., 2012, Lopes et al., 2018, Lopes et al., 2019, Papanikolaou et al., 2001, Papanikolaou et al., 2002a, Papanikolaou et al., 2002b, Papanikolaou et al., 2003, Yang et al., 2012).

*Y. lipolytica,* grown in varied hydrophobic media, produces important nutritional components such as trace minerals, amino acids, including essential amino acids (e.g. lysine and methionine), peptides, or fats, especially mono-unsaturated fatty acids (MUFAs) and saturated high added-value lipids like cocoa-butter equivalents. Hence, such the yeast is also called nutritional yeast. Noteworthy, *Y. lipolytica* can accumulate lipids intracellularly up to ≥40% of its cell dry weight or produce 30–50% protein of dried biomass (Bellou et al., 2016, Beopoulos et al., 2011, Dourou et al., 2018, Drzymała et al., 2020, Jach et al., 2017, Juszczyk et al., 2013, Lopes et al., 2018, Lopes et al., 2019, Papanikolaou et al., 2001, Papanikolaou et al., 2003, Rywińska et al., 2013). Similar to animal cells, *Y. lipolytica* is also capable of vitamin B12 assimilation into its cells from biofuel waste acting as a substrate (Jach et al. 2020b).

*Y. lipolytica* is safe and non-pathogenic to humans, and its strains appear in many foods as living cells (e.g. in cheese, mayonnaise and meat) that rarely cause opportunistic infections, exclusively in patients with compromised immunity and those with catheters. Hence, the U.S. FDA has granted the “Generally Regarded as Safe (GRAS)” status to several production processes using *Y. lipolytica* (Groenewald et al., 2013, Zieniuk and Fabiszewska, 2019). In this regard, dried and heat-killed nutritional yeast cells can be added as a cheap supplement to the regular human diet to help in solving the problem of food deficiency in rapidly growing populations, especially in developing countries like India (Jach et al., 2017, Jach and Serefko, 2018, Kennedy, 2015). Additionally, the nutritional yeast biomass is obtained very fast (in comparison with the growth of plants or animals), from a relatively small area and regardless of the weather.

At present, *Y. lipolytica* is used as the nutritional biomass for livestock feeding and as a biotechnological production host for various substances for pharmaceutical, industrial and bioremediation processes (Groenewald et al., 2013). In 2010, the European Feed Manufacturers’ Federation authorized the *Y. lipolytica* biomass obtained from biofuel waste to be used as a feed additive. In turn, in 2019, the European Food and Safety Authority (EFSA) has authorized the use of dried and heat-killed *Y. lipolytica* biomass as a novel food in dietary supplements intended for the general population above 3 years of age (EFSA, 2019). The *Y. lipolytica* biomass cultivated in biofuel waste has previously been studied with regard to amino acids, single cell protein (SPC) and bioavailable vitamin B12 for human consumption (Jach et al., 2020b, Jach et al., 2017). However, there is hardly any information about the concentrations of other B vitamins in *Y. lipolytica* biomass in literature.

In contrast, conventional yeast, *Saccharomyces cerevisiae,* is well-known for its high content of water-soluble B vitamins and its use as dietary supplements for humans. Most wild-type strains of the yeast are prototrophic for all B vitamins. Apart from the *de novo* synthesis of these vitamins, a number of them (e.g. vitamin B12) can also be taken up from the growth substrate (Izah et al., 2019, Paalme et al., 2014). Thiamine, riboflavin, pyridoxine, biotin and folic acid have specific and catalytic functions as coenzymes that are involved in carbohydrate, fats, and protein metabolism. For this reason, the adequate levels of these vitamins are essential for an optimal physiological and neurological functioning of the human body. In fact, insufficient amounts of B vitamins are frequently found in the diets of the populations of developed countries (Kennedy, 2015).

The purpose of the presented study was to assess whether the amino acids and protein enriched biomass of *Y. lipolytica* cultivated in biofuel waste could serve as a source of vitamins B1, B2, B6, B7 and B9.

## 2 Materials and methods

### 2.1 Microbial strains

During the research, we used the wild-type *Yarrowia lipolytica* A-101 strain provided by Skotan S.A., Poland. The reference *Yarrowia lipolytica* ATCC 9793 strain, provided by LGC Standards, was included in some experiments.

### 2.2 Production of biomass, biomass harvesting, and variations of fermentation parameters

*Y. lipolytica* strains were cultivated in two culture media, i.e. the standard laboratory YPD medium (Difco) and the industrial SK medium as previously described (Jach et al. 2017). The SK medium is a waste formed during biofuel (biodiesel) production. Biofuel is made through chemical reaction of vegetable oil with ethanol producing fatty acid esters (long-chain alkyl (methyl, ethyl, or propyl) esters). Therefore, biofuel waste containing a mixture of vegetable oils, degumming and glycerol fractions (2–7% wt/wt) were used as carbon and energy sources. Degumming contains mainly phosphoric acid derivatives associated with fats and protein as well as free plant fats (up to 10%), protein (up to 10%) and ash (up to 5%). The SK medium also contains some amount of B vitamins. Mean concentration of these vitamins in 100 ml of this medium is as follows: 0.9 mg of thiamine; 3.65 mg of riboflavin; 3.38 mg of pyridoxine, 138 µg of folic acid and 6.2 µg of cyanocobalamin. The SK medium was provided by Skotan S.A., Poland. For *Y. lipolytica* cultivation, the biofuel production waste was replaced with a partially refined, desalinated, and methanol-free by-product from biodiesel manufacture (delivered from Lotos Group Refineries, Poland to Skotan S.A.). Two culture experimental conditions were used during the study: 1) a variable temperature (from 20 °C to 30 °C) and a constant pH (6.0); 2) a constant temperature (30 °C) and a variable pH (from 4.0 to 7.0). The pH was adjusted to required values by adding 1 M NaOH or 1 M NaCl, respectively. For obtaining vitamin B2, the media were additionally supplemented with a mixture of hypoxanthine (1 mg/L) and threonine (50 or 100 mg/L). For obtaining vitamin B7, the media were also supplemented with pimelic acid (2 or 4 g/L) alone or a mixture of pimelic acid (2 or 4 g/L) and L-alanine (1 or 2 g/L). The media were prepared and sterilized, and *Y. lipolytica* strains were cultured in the Erlenmeyer flasks (150 ml) and in a biofermentor (100 L) as previously described (Jach et al., 2017). Briefly, the inoculation cultures were grown for 2-days with initial OD_650_ around 0.15 followed by cultivation in shaking flask or biofermentor. On the laboratory scale, the cultures were grown in the flask shook at 200 rpm in an incubator shaker for 12 or 18 h. On the pilot scale, the cultures of *Y. lipolytica* A-101 strain were cultivated in SK medium in biofermentors at temperature of 30 °C, pH 5.0 with mechanical agitation and 40% oxygenation per 12 h. After cultivation, biomass was separated from culture media by centrifugation at 8000*g* for 15 min in order to pellet down the yeast cells and washed three times with sterile water. The biomass obtained from the biofermentor was transferred into a tumble dryer and dried at 165–175 °C for 1 h; this yielded dried biomass called the *Yarrowia* powder.

### 2.3 Determination of the concentration of B vitamins in wet and dry yeast biomass

The analyses of B vitamins in the SK medium (biofuel waste), wet and dry biomasses were carried out using recommended microbiological assays with a VitaFast® B1 Microbiological microtiter plate test for determination of vitamin B1 (Thiamine) (R-Biopharm), as described by AOAC Official Methods 960.46 (AOAC, 2006a, AOAC, 2006b); a VitaFast® B2 Microbiological microtiter plate test for determination of vitamin B2 (Riboflavin) (R-Biopharm), as described by AOAC Official Methods 960.46 (AOAC, 2006a, AOAC, 2006b) and AOAC 940.33 (AOAC, 2006a, AOAC, 2006b); a VitaFast® B6 Microbiological microtiter plate test for determination of vitamin B6 (Pyridoxine) (R-Biopharm), as described by AOAC Official Methods 960.46 (AOAC, 2006a, AOAC, 2006b), AOAC 961.15 (AOAC, 1975) and AOAC 985.32 (AOAC, 1988); and a VitaFast® B7 Microbiological microtiter plate test for determination of vitamin B7 (Biotin) (R-Biopharm), as described by AOAC Official Methods 960.46 (AOAC, 2006a, AOAC, 2006b); and a VitaFast® B9 Microbiological microtiter plate test for determination of vitamin B9 (folic acid) (R-Biopharm), as described by AOAC Official Methods 960.46 (AOAC, 2006a, AOAC, 2006b), AOAC 992.05 (AOAC, 2016) and AOAC 944.12 (AOAC, 1996). The microbiological quantification of the vitamins was performed according to manufacturer’s instructions.

### 2.4 Statistical analysis of data

All data are expressed as a mean ± SD (standard deviation) of three independent experiments. The differences between the concentrations of B vitamins in the biomass obtained in the different conditions were compared to the *Y. lipolytica* A-101 strain cultivated in the YPD medium at 30 °C and pH 6.0, with two-sided student’s *t*-test, using Statistica software version 12.0. The *P* value < 0.05 was considered statistically significant.

## 3 Results

### 3.1 Influence of culture conditions on vitamin B1 concentration in the biomass of *Y. lipolytica*

**Fig. 1:**
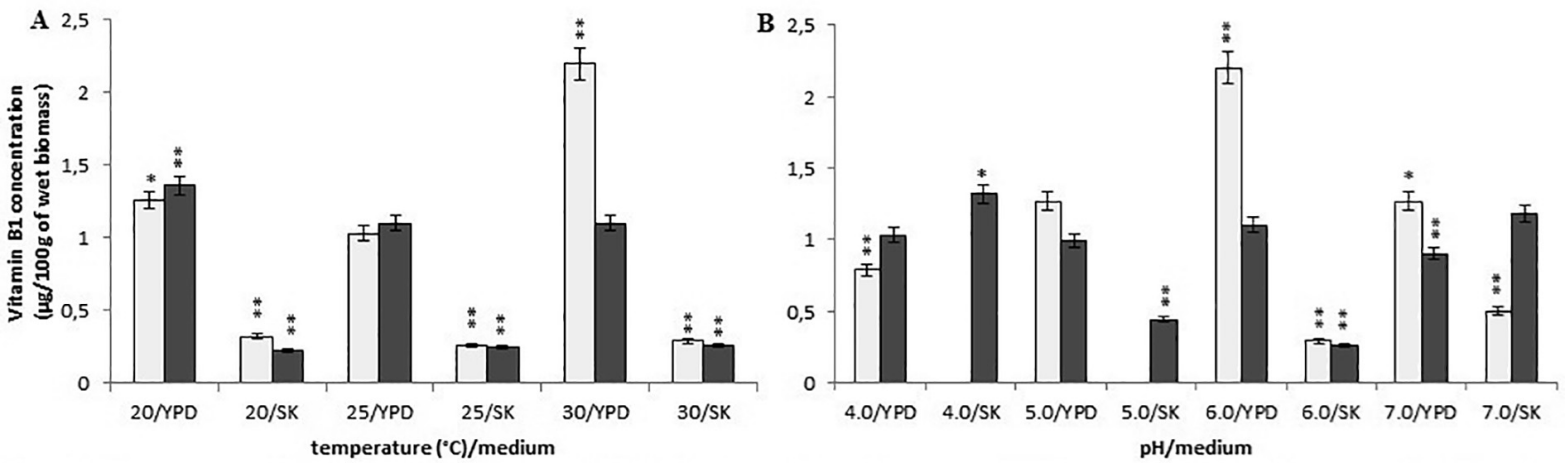
Concentration of vitamin B1 in the wet biomass of *Y. lipolytica* strains in various culture conditions and media on a laboratory scale: (A) temperature effect at pH 6.0; (B) pH effect at 30 °C; symbols (for A, B panels): *Y. lipolytica* ATCC 9793 (blank squares); *Y. lipolytica* A-101 (filled squares); *P < 0.05 and **P < 0.01 indicate a significant difference between the concentrations of vitamin B1 in the biomass obtained in the different conditions, compared to the *Y. lipolytica* A-101 strain cultivated in the YPD medium at 30 °C and pH 6.0.

The same levels of vitamin B1 content in the *Y. lipolytica* A-101 strain at pH 6.0 and irrespective of the temperature (from 20 °C to 30 °C) (mean 0.25 ± 0.15 mg/100 g of wet biomass) in the cultures in the SK medium (biofuel waste) were observed (Fig. 1A). However, in the same conditions, the *Y. lipolytica* A-101 cultured in the YPD medium contained a much higher level of vitamin B1, i.e. above 1.0 mg/100 g of wet biomass. Interestingly, the highest concentration of thiamine (1.36 ± 0.07 mg/100 g of wet biomass) was found in this strain at the lowest incubation temperature of 20 °C in the YPD medium. This difference was statistically significant (*P* < 0.01). In contrast, the biomass of the reference *Y. lipolyti*ca ATCC 9793 stain contained the highest vitamin B1 concentration (2.20 ± 0.11 mg/100 g of wet biomass) when the yeast was cultivated at the temperature of 30 °C in the YPD medium. Similar to *Y. lipolytica* A-101, the reference strain, grown in the SK medium, regardless of the applied temperature (from 20 °C to 30 °C), exhibited almost the same levels of vitamin B1 content in its wet biomass (a mean value of 0.29 ± 0.07 mg/100 g) (Fig. 1A).

In the case of the *Y. lipolytica* A-101 strain grown in the SK medium (biofuel waste) at 30 °C (pH from 4.0 to 7.0), decreasing pH to 4.0 influenced significantly the increase of thiamine concentration (1.32 ± 0.07 mg/100 g of wet biomass) (Fig. 1B). The difference was statistically significant (*P* < 0.05). The *Y. lipolytica* ATCC 9793 strain did not grow at low pH (4.0 or 5.0) in the SK medium (biofuel waste), in contrast to the growth in the YPD medium. For the reference strain, the observed changes in pH (4.0, 5.0 or 7.0) did not trigger an increase in vitamin B1 concentration (Fig. 1B). Then, regardless of the culture conditions, uptake and accumulation of thiamine in biomass of *Y. lipolytica* strains was observed.

### 3.2 Influence of culture conditions and addition of hypoxanthine and threonine on vitamin B2 content in *Y. lipolytica* biomass

**Fig. 2:**
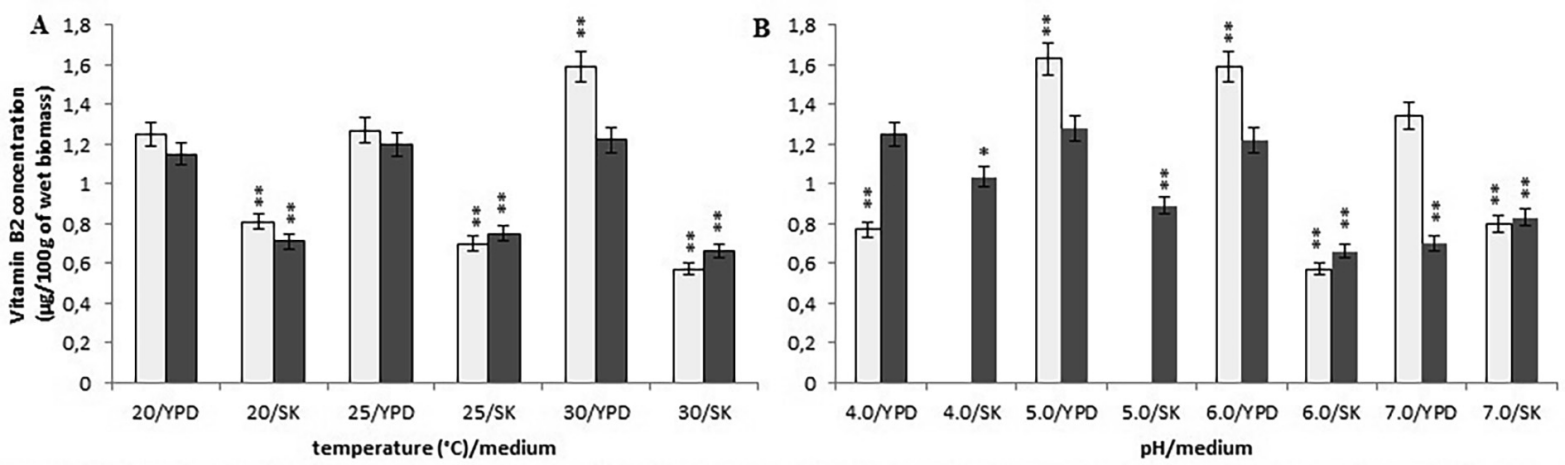
Concentration of vitamin B2 in the wet biomass of *Y. lipolytica* strains in various culture conditions and media on a laboratory scale: (A) temperature effect at pH 6.0; (B) pH effect at 30 °C; symbols (for A, B panels): *Y. lipolytica* ATCC 9793 (blank squares); *Y. lipolytica* A-101 (filled squares); *P < 0.05 and **P < 0.01 indicate a significant difference between the concentrations of vitamin B2 in the biomass obtained in the different conditions, compared to the *Y. lipolytica* A-101 strain cultivated in the YPD medium at 30 °C and pH 6.0.

In both media, i.e. the YPD and SK media with constant pH 6.0, temperature changes from 20 °C to 30 °C did not influence significantly vitamin B2 concentration in the *Y. lipolytica* A-101 cultures (Fig. 2A). The results were comparable to all fermentation samples (a mean value of 0.71 ± 0.05 mg/100 g of wet biomass) at these temperatures, while cultivated in the SK medium (biofuel waste). However, at these temperatures, the biomass of the *Y. lipolytica* A-101 strain when was cultured in the YPD medium contained a higher content of vitamin B2, with a mean value of 1.21 ± 0.05 mg/100 g of wet biomass. In contrast, the *Y. lipolyti*ca ATCC 9793 strain showed the highest content of vitamin B2 (1.59 ± 0.08 mg/100 g of wet biomass) when grown at the temperature of 30 °C in the YPD medium.

The *Y. lipolytica* A-101 strain grown in the SK medium at a constant temperature of 30 °C, in low pH 4.0 and 5.0 had a significantly higher vitamin B2 concentration (1.04 ± 0.05 mg/100 g and 0.89 ± 0.02 mg/100 g of wet biomass, respectively) comparing to higher pH (6.0 and 7.0) (0.66 ± 0.03 mg and 0.70 ± 0.04 mg/100 g of wet biomass, respectively) (Fig. 2B). These differences were statistically significant (*P* < 0.05 or *P <* 0.01). For this strain grown in the YPD medium, pH changes ranging from 4.0 to 6.0 did not influence riboflavin concentration, while pH 7.0 caused a significant decrease in vitamin B2 content (Fig. 2B). The analyses also revealed that the temperature of 30 °C and pH 6.0 and 5.0 were the optimal conditions for the cultivation of *Y. lipolytica* ATCC 9793 in the YPD medium, in terms of vitamin B2 content (1.63 ± 0.08 mg and 1.59 mg ± 0.08/100 g of wet biomass, respectively).

**Fig. 3:**
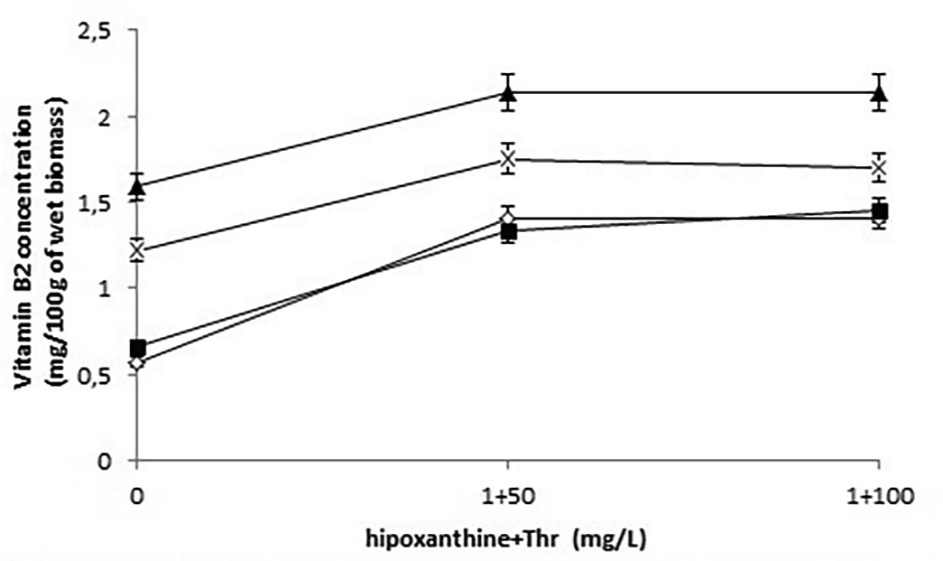
Concentration of vitamin B2 in the wet biomass of *Y. lipolytica* strains after the addition of hypoxanthine (1 mg/l) and threonine (50 or 100 mg/l) (30 °C, pH 6.0); symbols: *Y. lipolytica* ATCC 9793 grown in the SK medium (blank rhombus); *Y. lipolytica* ATCC 9793 grown in the YPD medium (filled triangle); *Y. lipolytica* A-101 grown in the SK medium (filled square); *Y. lipolytica* A-101 grown in the YPD medium (cross).

Apart from the actual growth conditions, the supplementation of various precursors such as hypoxanthine and threonine to a medium may affect the production of vitamin B2 (O’Toole and Kun, 2013). The addition of a mixture of hypoxanthine (1 mg/L) and threonine (50 or 100 mg/L) to the media at the temperature of 30 °C and pH 6.0 resulted in an increased riboflavin concentration in the biomass of both strains, especially at low threonine concentrations (0 to 50 mg/L) (Fig. 3). Riboflavin content increased by 45% and 75% in biofuel waste (SK medium) and the YPD medium, respectively (Fig. 3). These differences were statistically significant (*P* < 0.05). A further increase in threonine concentrations (up to 100 mg/L) with a constant concentration of hypoxanthine (1 mg/L) did not cause statistically significant changes in vitamin B2 levels in the yeast biomass.

### 3.3 Influence of culture conditions on vitamin B6 concentration in *Y. lipolytica* biomass

**Fig. 4:**
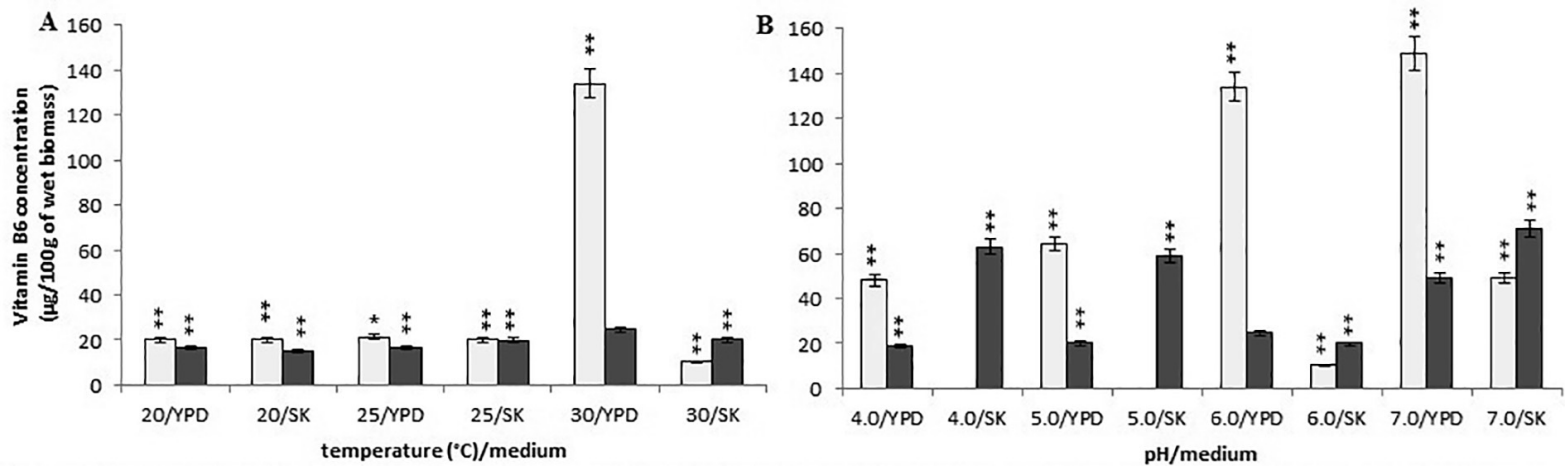
Concentration of vitamin B6 in the wet biomass of *Y. lipolytica* strains in various culture conditions and media on a laboratory scale: (A) temperature effect at pH 6.0; (B) pH effect at 30 °C; symbols (for A, B panels): *Y. lipolytica* ATCC 9793 (blank squares); *Y. lipolytica* A-101 (filled squares); *P < 0.05 and **P < 0.01 indicate a significant difference between the concentrations of vitamin B6 in the biomass obtained in the different conditions, compared to the *Y. lipolytica* A-101 strain cultivated in the YPD medium at 30 °C and pH 6.0.

For the *Y. lipolytica* A-101 strain cultivated in the SK medium (biofuel waste) at pH 6.0, we observed the same levels of pyridoxine content at higher temperatures (from 25 °C to 30 °C) (a mean value of 19.9 ± 0.15 mg/100 g of wet biomass) while at low temperature of 20 °C (14.7 ± 0.15 mg/100 g of wet biomass) (Fig. 4A). In turn, the *Y. lipolytica* ATCC 9793 strain grown in biofuel waste resulted in a significantly lower content of vitamin B6 when cultivated at 30 °C, compared to the vitamin concentration obtained at the temperature range between 20 °C and 25 °C (Fig. 4A). However, when the reference strain was cultured in the YPD medium at the temperature of 30 °C, the highest content of pyridoxine was observed (134 ± 1.71 µg/100 g of wet biomass) (Fig. 4A).

For the *Y. lipolytica* A-101 strain grown in the SK medium (biofuel waste) at the temperature of 30 °C (pH from 4.0 to 7.0), a decreased pH (4.0 and 5.0) resulted in a 150% increase in the concentration of pyridoxine (a mean value of 61.5 ± 2.50 µg/100 g of wet biomass), compared to vitamin B6 content in the yeast strain biomass obtained at 30 °C and pH 6.0 in both the YPD and SK media (Fig. 4B). A comparable level of pyridoxine concentration was also found in the biomass of *Y. lipolytica* A-101 grown in biofuel waste (SK medium) at 30 °C and pH 7.0 (71 ± 3.55 µg/100 g of wet biomass). Interestingly, using the YPD medium for the A-101 strain growth did not cause an increase in the concentration of vitamin B6, which was detected in the case of other vitamins under analysis. At the same time, the wet biomass of the reference *Y. lipolyti*ca ATCC 9793 strain exhibited the highest vitamin B6 concentration when grown in the YPD medium at a temperature of 30 °C and pH 7.0 (149 ± 1.76 µg/100 g of wet biomass, respectively) (Fig. 4B).

### 3.4 Influence of culture conditions on vitamin B7 content in *Y. lipolytica* biomass

**Fig. 5:**
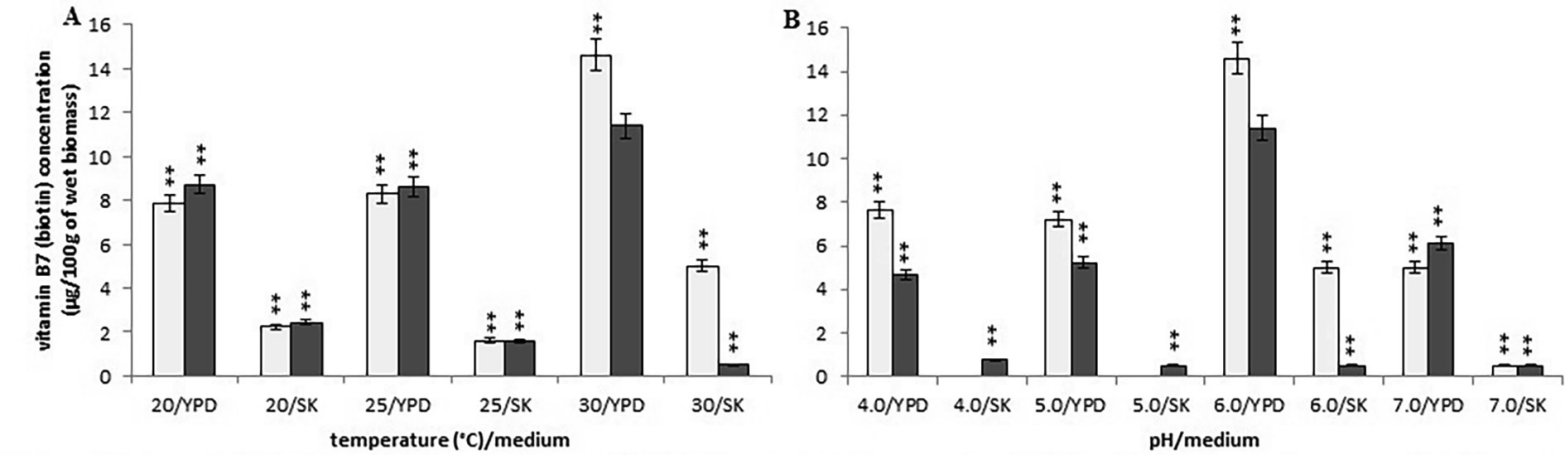
Concentration of vitamin B7 in the wet biomass of *Y. lipolytica* strains in various culture conditions and media on a laboratory scale: (A) temperature effect at pH 6.0; (B) pH effect at 30 °C; symbols (for A, B panels): *Y. lipolytica* ATCC 9793 (blank squares); *Y. lipolytica* A-101 (filled squares); *P < 0.05 and **P < 0.01 indicate a significant difference between the concentrations of vitamin B7 in the biomass obtained in the different conditions, compared to the *Y. lipolytica* A-101 strain cultivated in the YPD medium at 30 °C and pH 6.0.

The *Y. lipolytica* A-101 biomass contained the highest comparable concentration of vitamin B7 when was cultured in the SK medium (biofuel waste) at temperatures of 20 °C and 25 °C, pH 6.0 (2.50 ± 0.19 µg/100 g and 2.44 ± 0.12 µg/100 g of wet biomass, respectively) (Fig. 5A). In contrast, the strain grown in the YPD medium had the highest content of biotin at the temperature of 30 °C (11.40 ± 0.57 µg/100 g of wet biomass). The *Y. lipolyti*ca ATCC 9793 strain grown in the YPD medium at the temperature of 30 °C also contained the highest vitamin B6 concentration (14.60 ± 0.73 µg/100 g of wet biomass) (Fig. 5A). In the case of the *Y. lipolytica* A-101 strain grown in the SK medium at the constant temperature of 30 °C, pH changes (4.0, 5.0 or 7.0) did not influence vitamin B7 concentration (Fig. 5B).

We also tested the effect of the addition of biotin precursors such as pimelic acid (2 or 4 g/L) and a mixture of pimelic acid (1 or 500 g/L) and alanine (1 or 2 g/L) to the media (Gadre and Rao, 1975), but they had no influence on the vitamin B7 contents in the biomass of both yeast strains grown in the YPD medium or on the *Y. lipolytica* ATCC 9793 strain cultured in supplemented biofuel waste. The biotin levels were comparable in all cultures (data not shown). A significant influence on the concentration of vitamin B7 was observed when the *Y. lipolytica* A-101 strain was cultivated in the SK medium (biofuel waste), supplemented with a low concentration of pimelic acid (2 g/L) alone (2.07 ± 0.10 µg/100 g of wet biomass), as well as with the mixture of pimelic acid (1 g/L) and alanine (1 g/L) (1.91 ± 0.10 µg/100 g of wet biomass). It resulted in up to 25% more of vitamin B7. However, the productivity of biotin by the yeast strains cultivated in supplemented biofuel waste (SK medium) was still on the lower level in comparison with uptake and the accumulation of this vitamin by *Y. lipolytica* strains grown in standard YPD medium.

### 3.5 Influence of culture conditions on vitamin B9 content in *Y. lipolytica* biomass

**Fig. 6:**
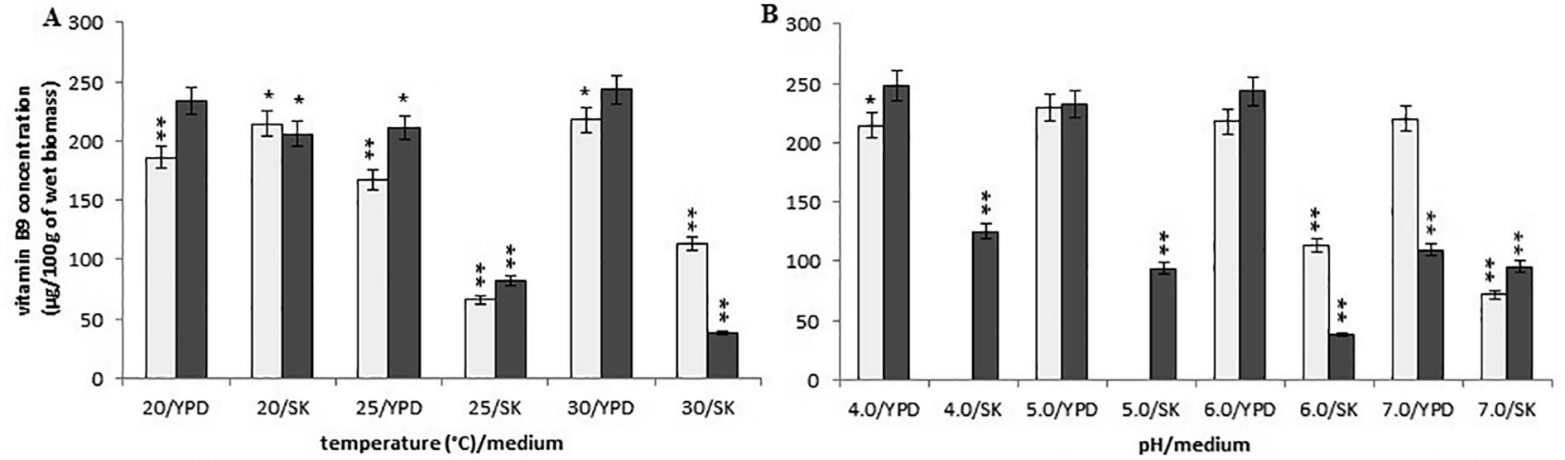
Concentration of vitamin B9 in the wet biomass of *Y. lipolytica* strains in various culture conditions and media on a laboratory scale: (A) temperature effect at pH 6.0; (B) pH effect at 30 °C; symbols (for A, B panels): *Y. lipolytica* ATCC 9793 (blank squares); *Y. lipolytica* A-101 (filled squares); *P < 0.05 and **P < 0.01 indicate a significant difference between the concentrations of vitamin B9 in the biomass obtained in the different conditions, compared to the *Y. lipolytica* A-101 strain cultivated in the YPD medium at 30 °C and pH 6.0.

In both media, i.e. YPD and SK, at constant pH 6.0 and the variable temperature (ranging 20 °C-30 °C), the cultivation of *Y. lipolytica* A-101 at the temperature of 20 °C resulted in high levels of vitamin B9 concentration (234 ± 11.70 µg/100 g and 206 ± 10.30 µg /100 g of wet biomass, respectively) (Fig. 6A). However, at the temperature of 30 °C in the YPD medium, the *Y. lipolytica* A-101 strain biomass contained almost the same concentration of folic acid, with a mean value of 246 ± 12.30 µg/100 g of wet biomass. The *Y. lipolyti*ca ATCC 9793 strain showed the highest concentration of vitamin B9 when grown at the temperature of 30 °C (pH 6.0) in the YPD medium (218 ± 10.88 µg /100 g of wet biomass) or when cultivated at the temperature of 20 °C in the SK medium (214 ± 10.70 µg /100 g of wet biomass).

In the case of the *Y. lipolytica* A-101 strain grown in the SK medium at 30 °C, pH changes significantly influenced folic acid content (125 ± 6.25 µg, 94 ± 4.70 µg and 95 ± 4.75 µg /100 g of wet biomass in pH 4.0, 5.0 or 7.0, respectively) (Fig. 6B). However, in the reference *Y. lipolytica* ATCC 9793 strain grown in the YPD medium, pH changes (4.0, 5.0 or 7.0) did not influence vitamin B9 concentration.

### 3.6 Concentration of vitamins B1, B2, B6, B7 and B9 in the *Yarrowia* powder

In this study, we also determined the content of thiamine, riboflavin, pyridoxine, biotin and folic acid in the dried *Y. lipolytica* A-101 biomass (the *Yarrowia* powder) obtained through standard production (in biofermentors using biofuel waste called SK medium as carbon and energy source) of seven independent batches on a pilot plant scale. The *Yarrowia* powder was an amorphous hygroscopic beige-coloured powder with a slight yeast odour. The concentration values of vitamins B in the *Yarrowia* powder are given in Table1. In accordance with Regulation 1169 (2011), the daily nutrient reference values (NRVs) for adults are also presented in the Table 1. Therefore, 100 g of the *Yarrowia* powder was adequate to completely cover the daily intake of thiamine, riboflavin, pyridoxine and folic acid. However, the concentration of biotin in the *Yarrowia* powder (100 g of dry cell weight) covered about 50% of the NRVs determined for that vitamin.

**Table 1:** Concentration of protein and B vitamins in the dried *Yarrowia lipolytica* A-101 biomass (the *Yarrowia* powder) obtained after culturing in the SK medium (biofuel waste) on pilot plant scale. Conditions of cultivation: 100 L, 30 °C, pH 5.0, 40% oxidation, 12 h.

Batch number^1^	Protein content (% of dry weight)^2^	Concentration of vitamin					
		B1	B2	B6	B7	B9	B12^3^
		(mg/100 g of dry weight ± SD)			(µg/100 g of dry weight ± SD)		
1	49.3	1.22 ± 0.04	2.90 ± 0.15	5.62 ± 0.28	62.00 ± 3.10	300 ± 15.00	7.4
2	41.9	2.23 ± 0.68	6.24 ± 0.31	6.50 ± 0.33	14.15 ± 0.71	233 ± 11.70	9.9
3	42.6	2.23 ± 0.69	6.90 ± 0.35	7.40 ± 0.37	9.32 ± 0.47	184 ± 9.20	9.6
4	43.7	2.01 ± 0.26	5.38 ± 0.27	5.40 ± 0.27	10.02 ± 0.50	198 ± 9.90	8.6
5	44.8	0.68 ± 0.06	5.42 ± 0.27	2.88 ± 0.14	13.00 ± 0.65	330 ± 16.50	7.9
6	42.0	0.68 ± 0.02	5.13 ± 0.26	2.74 ± 0.14	18.50 ± 0.68	245 ± 12.30	8.7
7	41.9	0.75 ± 0.05	5.10 ± 0.26	3.84 ± 0.19	13.00 ± 0.83	251 ± 12.60	8.7
Mean	45.6	1.34	5.30	4.91	19.99	248.70	8.7
NRVs^4^	50	1.10	1.40	1.40	50.00	200.00	2.5

1 Each batch was obtained from a different and independent biofermentor culture.

2 Jach et al., 2017.

3 Jach et al., 2020a.

4 Regulation 1169/2011.

## 4 Discussion

In the presented work, we examined the effect of the culture conditions and used media on the B vitamins (thiamine, riboflavin, pyridoxine, biotin and folic acid) rate production in *Y. lipolytica* strains. The data presented here demonstrates that, regardless of the culture conditions, both the *Y. lipolytica* strains included these vitamins in their biomass. However, the concentration of the vitamins in yeast biomass depended, to some extent, on the strains and culture conditions. *Y. lipolytica* strains during uptaking and accumulating B vitamins exhibit different sensitivities to temperature and pH. The trial evaluated the effect of different values of temperature (from 20 °C to 30 °C) and pH (from 4.0 to 7.0) on concentration of B vitamins in yeast biomasses. The highest levels of vitamin B1, B2, B6 and B9 in the fermentation carried out by the *Y. lipolytica* A-101 strain were obtained when it grown in the SK medium (biofuel waste) at a temperature of 30 °C and pH 4.0, although promising results were also obtained at pH 5.0 (Fig. 1, Fig. 2, Fig. 4, Fig. 6). These differences were not statistically significant. In these conditions, only the concentration of vitamin B7 was not at its highest value (Fig. 5). However, the results suggest that the assimilation of B vitamins by the yeast depended primarily on the sources of carbon and nitrogen. Moreover, in the case of the cultivation in the non-conventional fatty medium such as biofuel waste (SK medium), decreasing the environmental factor like pH (below 5.0) is enough to cause significant increase of uptake and the accumulation of thiamine and riboflavin by *Y. lipolytica* A-101 strain to the level of these vitamins concentration obtained from biomass of *Y. lipolytica* cultivated in standard YPD medium. Additionally, our previous experimental and statistical studies on amino acids and protein production revealed that the temperature of 30 °C and pH 5.0 provided more suitable cultivation conditions for *Y. lipolytica* A-101 in biofuel waste (SK medium) than pH 6.0 at the same temperature value (Jach et al., 2017, Jach et al., 2020a). Using the statistical optimalization methods of fermentation parameters and media, we have shown that utilizing biofuel waste (SK medium), for obtaining amino acids and SPC-enriched biomass of *Y. lipolytica*, required a precise control of only one parameter, i.e. the pH level. While the cultures were cultivated in the YPD medium, the highest values of SPC were closely related to both the temperature and pH of the yeast cultivation (Jach et al., 2020a). Additionally, it was also shown that the culture conditions of 30 °C and pH 5.0 were sufficient to yield the *Y. lipolytica* A-101 biomass rich in amino acids and vitamin B12 when the yeast was cultivated in biofuel waste (SK medium) (Jach et al., 2020b). Noteworthy, culture conditions such as temperature and pH also strongly affect the activity of lipases that *Y. lipolytica* produces to break down fats (Dominguez et al., 2010, Lopes et al., 2019, Papanikolaou et al., 2002a, Papanikolaou et al., 2002b, Papanikolaou et al., 2007, Saygün et al., 2014, Thevenieau et al., 2010). The maximum activity of *Y. lipolytica* lipases was found at the temperature between 30 and 40 °C and at pH 5.0 while a significant decrease in stability of these enzymes was seen at pH values over 6.5 (Dominguez et al., 2010, Lopes et al., 2019). Therefore, amino acids, protein and vitamin B1, B2, B6, B9 and B12 production occurs under the same conditions in which lipases work most efficiently. Furthermore, the medium pH and the incubation temperature significantly influenced the accumulation of lipids by *Y. lipolytica* during the primary anabolic growth when cultivated in fatty substrates (Papanikolaou et al., 2002a, Papanikolaou et al., 2003, Papanikolaou et al., 2007, Zhao et al., 2016). In contrast, using adaptive laboratory evolution (ALE) strategies resulted in a 30% increase in lipid accumulation by the *Y. lipolytica* strain (Daskalaki et al., 2019).

As the results showed, the standard laboratory YPD medium consisting of peptone as sources of carbon, nitrogen, vitamins and minerals, yeast extract rich in vitamin B complex, and stimulating bacterial growth factors, was the much more adequate substrate than biofuel waste (SK medium) to obtain B vitamin-enriched yeast biomass. Paalme et al. (2014) have shown that it is not strictly required to add these vitamins to the culture of *S. cerevisiae* to obtain B vitamin-enriched yeast biomass. However, the addition of these vitamins to the medium was found to be increasing the productivity and accumulation of the B vitamins. The uptake of these vitamins from the environment would allow yeast cells to save intracellular resources of B vitamins for other biosynthesis processes. Hence, we observed higher concentrations of vitamins B1, B2, B6, B7 and B9 in the biomass of yeast cultured in the YPD medium, compared to the SK medium (biofuel waste). However, substrates such as glucose, peptone and yeast extract cannot be used freely in mass production as they are too expensive for the production of desired products (Katre et al, 2012). In this respect, the production of nutritional yeast biomass using the easily available inexpensive wastes as carbon and energy sources (e.g. biofuel waste), is desired by the industry in the broad sense. In fact, waste biodegradation is regarded as particularly relevant for environmental protection (Katre et al., 2012, Saygün et al., 2014, Vasiliadou et al., 2018, Tzirita et al., 2018). Noteworthy, a simple addition of an oil substrate (i.e. waste cooking oil) to the growth medium induces a significant increase in the production of extracellular lipases by *Y. lipolytica* compared to oil-free cultures (Dominguez et al., 2010, Lopes et al., 2019). This is supported by the fact that the yeast cultivated in fatty wastes at first accumulates storage lipids. When extracellular carbon sources exhaust, the storage lipids allow sustaining the yeast growth by using them for various metabolic activities and as an energy source (Daskalaki et al., 2019, Dourou et al., 2018). Furthermore, in nitrogen starvation (irrespective of non-oleaginous and oleaginous conditions), the significant degradation of storage lipids was found to coincide with the production of fat-free biomass and an increase in protein concentration (Bellou et al., 2016, Daskalaki et al., 2019, Dourou et al., 2018). Additionally, we have found that fat-free, amino acids and protein enriched *Y. lipolytica* biomass also contained water-soluble B vitamins. Therefore, the yeast biomass could be introduced as aid to improve food quality and health status of humans in developed countries.

In addition to the actual growth conditions, O’Toole and Kun (2013) postulated that the supplementation of various vitamin precursors to the media may affect their biosynthesis by *S. cerevisiae*. These authors reported that an increase in riboflavin content was induced by the addition of hypoxanthine and threonine which were necessary for efficient biosynthesis of vitamin B2. In our research, we confirmed that the addition of riboflavin agents significantly increased vitamin B2 concentration in the biomass of *Y. lipolytica* strains, especially at low threonine concentrations (50 mg/L). Riboflavin content increased by 45% and 75% when the yeast strains were grown in biofuel waste (SK medium) and the YPD medium, respectively. These results suggest that while supplementation of the culture with riboflavin is not strictly required, adding riboflavin precursors such as hypoxanthine and threonine in optimal concentrations can increase the productivity of vitamin B2 by the yeast. We also examined the effect of an addition of biotin precursors, such as pimelic acid and alanine, on this vitamin content in yeast biomass (Gadre and Rao, 1975). The supplementation of these factors to biofuel waste (SK medium) led to an increase in vitamin B7 content only in case of the *Y. lipolytica* ATCC A-101 biomass (25%). However, the production of biotin was still on very low level. The results suggest that the concentration of biotin in the feeding medium was crucial for its assimilation in yeast biomasses. The vitamin B7 content in YPD medium contributed to increase of the accumulation rates (approximately sixfold) in comparison with amount of biotin obtained from biomass of *Y. lipolytica* cultivated in biofuel waste (SK medium) at the same conditions.

Drying at high temperatures kills the yeast and destroys its cell walls, while releasing the nutritious contents and improving the digestibility of the biomass (Adedayo et al. 2011). In this respect, the bioavailability of dietary B vitamin-enriched yeast biomass is an argument for its inclusion as a diet supplementation for both humans and animals (Izah et al., 2019, Paalme et al., 2014). Therefore, we examined the concentration of vitamins B1, B2, B6, B7 and B9 in the dried biomass of *Y. lipolytica* A-101 cultivated in biofuel waste (SK medium). The adequate nutrition of B vitamins is particularly important in developed countries where deficiencies in essential micronutrients exist. As B vitamins are water-soluble, their excess is generally excreted in urine. It means that doses much higher than the recommended daily intake (NRVs) are safe. Simultaneously, the intake of B vitamins must be more consistent than that of the fat soluble ones (Kennedy, 2015). Previously, we found that 100 g of the *Yarrowia* powder supplied the recommended daily intake of “complete” protein and vitamin B12 for adults (Jach et al., 2017, Jach et al., 2020b). This work showed that this quantity of the *Yarrowia* powder was also sufficient to completely cover the required NRVs for thiamine, riboflavin, pyridoxine and folic acid. However, biotin concentration in 100 g of the *Yarrowia* powder covered about 50% of the recommended daily intake.

## 5 Conclusions

According to the results presented in this paper, the *Yarrowia* powder can be regarded as a good source of vitamins B1, B2, B6 and B9. Additionally, our observations clearly indicate that biofuel waste is a promising raw material for the cultivation of the *Y. lipolytica* biomass which could be used in food products. The yeast biomass can be included as an addition to food, especially for people who avoid eating meat (e.g. following vegan and vegetarian diets) or live in poor regions and places with limited food supplies, in order to prevent the risk of deficiencies of B vitamins. Taken together, this data showed that the *Y. lipolytica* biomass could represent an important and useful component for human diet.

## 6 Ethics approval and consent to participate

Not applicable.

## 7 Consent for publication

Not applicable.

## 8 Availability of data and materials

The datasets generated for this study are available on request to the corresponding author.

## Funding

This work was supported by funds from the European Regional Development Fund under the Innovative Economy Operational Programme 2007–2013 [UDA-POIG-01.04.00–24-132/11–00].

## Authors' contributions

MEJ and AM designed the experiments; MEJ, ES, MJa, MJu and EK performed experimental research; MEJ and AM performed data analysis. MEJ wrote the manuscript text; MEJ, MJa, TB and AM edited the manuscript text. MJa, ES and AM critically revised the manuscript. All authors read and approved the final manuscript.

## Declaration of Competing Interest

The authors declare that they have no known competing financial interests or personal relationships that could have appeared to influence the work reported in this paper.
